# Howard Chang receives the 2024 ASCI/Stanley J. Korsmeyer Award

**DOI:** 10.1172/JCI180678

**Published:** 2024-04-01

**Authors:** Lisa R. Conti

The American Society for Clinical Investigation (ASCI) honors Howard Chang with the 2024 Stanley J. Korsmeyer Award. Dr. Chang (Figure 1) is recognized for his contributions to genome science through his invention of mapping technologies and his discoveries of long noncoding RNAs (lncRNAs). Dr. Chang is a professor of Dermatology and Genetics at Stanford University and belongs to the American Academy of Arts and Sciences, the National Academy of Sciences, and the National Academy of Medicine. He is also a Howard Hughes Medical Institute investigator, a recipient of the NAS award in Molecular Biology from the National Academy of Sciences, and Director of the Center for Personal Dynamic Regulomes, an NIH Center of Excellence in Genomic Science. Dr. Chang and his research group have developed methods for revealing regulators of transcriptional programs involved in cell identity and disease. The *JCI* recently spoke with Dr. Chang about his research career and impactful discoveries.

*JCI*: Tell us about your early exposure to science and how it started you on a path that involved research and medicine.

Chang: The summer after my freshman year in college I had the opportunity to work in a biochemistry lab at Harvard Medical School with the late Professor Christopher Walsh. I got exposed to rigorous scientific thinking and the possibility that scientific approaches uncover answers to deep biological questions. In that experience, I also met, for the first time, MD-PhD students. Knowing that it was possible for people to pursue both kinds of training together planted a seed for what I may want to do with my own training.

In the Walsh lab, we were trying to understand the mechanism of the immunosuppressive drug cyclosporine. Before cyclosporine, organ transplantation from one person to another had to be, basically, from an identical twin, because otherwise the immune system would reject the organ. This drug restrained the immune system so that the recipient would accept the other organ; later it was used to treat other kinds of autoimmune diseases. But people did not know how it worked. There was a race in the early 1990s where people were using it as a probe to find out the signaling mechanisms that turned out to involve T cells. I came into the lab thinking that I may want to pursue a career in medicine. During my undergraduate thesis, I had a chance to work on my own project on the same pathway targeted by cyclosporine. That was my first experience working on a research project and resulted in my first-author publication (1). Later I was able to join the Harvard/MIT MD-PhD Program.

*JCI*: Your early research work spanned biological topics, including transplantation and apoptosis. How did you come to settle on dermatology as a specialty in medicine?

Chang: During my PhD at MIT, I worked with Professor David Baltimore on some fundamental mechanisms of programmed cell death. It was a basic topic, and it could have applied to many fields. After completing my PhD research, I didn’t go back to medical school with any clearly preconceived notion that I was going to be in a particular specialty field. Dermatology really appealed to me because I felt that I could observe the biology of the different disease processes very directly. I also felt that there were many fundamental questions in the field of skin biology that were not answered yet. And so maybe I would have a niche to try to answer some of these questions.

*JCI*: When did you first suspect that long RNA transcripts from noncoding DNA had a regulatory role?

Chang: During my postdoctoral training, I worked with Pat Brown and David Botstein, who were pioneers in emerging genome-wide technologies to measure the activities of every gene in the body. This was the time when the human genome project had just reached completion, so, for the first time, it was possible to do these kinds of studies. I was trying to figure out what made skin from different parts of the body different. For example, we have hair on the top of our scalps, but we don’t have hair on our palms and soles. We were designing experiments to look at genes that were expressed only in skin cells from one part of the body but not the other (2). At the time, with microarray technology, you would take regions of DNA that were supposed to contain genes and put them on a glass slide in spots. A glass slide would have 20,000 spots and you could measure the expression of all these genes. It turns out that when the Human Genome Project was done, people realized that just 2% of the human DNA contained coding genes, and the other 98% was noncoding. Most people’s arrays did not contain anything that was noncoding, because it seemed like a waste of time and energy. But we did an experiment with a gene region we cared about (the *HOX* loci) (3). We said, let’s just put spots down for all the known genes and also include everything in between, as though the noncoding sequences would be a negative control. We did the experiment, but found all these regions were lighting up, meaning that the cells were making RNA. In addition, we found many RNAs that would be expressed only in certain skin cells from different body parts. These new RNAs had a regulated pattern of activity, suggesting that the signal was not some sort of noise. When we first saw it, I thought something was really off. When we did a complete unbiased analysis, we found more of these new RNAs and they were showing as much regulated expression as the known coding genes. There was such a huge signal, you couldn’t just ignore it. To prove to ourselves that this was not background, we made a single RNA and put it onto the array. Back in the day, microarrays were quite expensive. So anytime you got your hands on a small number of these, you were trying to do some sort of maximally informative experiment. To use a bunch of arrays just hybridizing a single RNA was a bit crazy, but we had to know if it was real.

The work on lncRNAs is still ongoing because we now know that there’s 60,000, or perhaps more, of these lncRNAs.

*JCI*: You are well known for developing the assay for transposase-accessible chromatin using sequencing (ATAC-Seq) with William Greenleaf. Tell us about the assay and how it shaped your work.

Yes, it’s pronounced ATAC-Seq like you’re attacking the DNA. The idea is that in every human cell there’s about two meters of DNA if it’s stretched out end to end. But it’s also all squished into a 10-μm nucleus, so only the parts of the DNA that the cell is using are accessible. Of course, all cells in the body have the same DNA, but skin cells are not the same as brain cells. And that is because the different cells are using different bits of information. In the corresponding cell types, there are corresponding controls of DNA that are open and accessible. This is one of the basic mechanisms that determines cell identity. ATAC-Seq can find the DNA switches that control when and where genes turn on and off by revealing the accessible, open chromatin, areas. We use a transposase enzyme to spray paint the DNA. In the past, people understood this principle about DNA accessibility, but the biochemical methods were laborious. You needed tens of millions of cells, and it would take days. Those kinds of requirements made it basically impossible to make measurements in clinical samples. It also meant growing cells up over weeks. By the time you amplify one cell into 10 million, you are not really studying the original anymore. I wanted to get that level of DNA-accessibility information directly from clinical materials. So, this method had to be much more sensitive compared with the prior state of the art. ATAC-Seq improved the sensitivity by about a million-fold (4). It was also about a hundred times faster. So, that was what made it possible for the first time to make these measurements directly in clinical samples. Later, we became able to make these measurements in single cells, and that also opened a new field of single-cell epigenetics.

*JCI*: You’ve published work on the connection between lncRNA and female-biased autoimmune disease. Can you explain this work to us?

Chang: This publication just came out and addresses a longstanding medical mystery; hopefully, we now have insight and perhaps a major answer (5). The observation is that four out of five patients with autoimmune disease are women. And that’s an average. For a fairly common disease like lupus, the ratio is nine to one, female to male. It is a really striking sex bias. A lot of these diseases are skin diseases, which I see as a practicing physician. In the past, people thought about different sex hormones or different sex chromosomes. Our study found that there’s a single RNA coming from the second X chromosome in women that is perhaps largely responsible. The single RNA, Xist, is a lncRNA involved in controlling gene activity. It basically shuts down the second X chromosome in female cells, and it only operates when there are two X chromosomes; that’s why it’s female specific. Because males have a single X and a Y chromosome, the males never express Xist. When female cells die and Xist RNA–protein complexes leak out, this RNA and all its protein partners can trigger the immune system. We generated a male mouse that expresses Xist, and it develops autoimmune disease at female levels. That proved that you don’t need female hormones. You don’t even need the rest of the second X chromosome — this RNA is an important driver.

*JCI*: You’ve done work on extrachromosomal DNA (ecDNA) and ecDNA hubs as it relates to cancer. Can you discuss this work?

We’re really interested in gene control coming from the noncoding part of the genome. This is a situation where there are cancer-causing genes that break off from chromosomes and turn into small circles. In the case of ecDNAs, the ecDNA expresses more than the exact same DNA sequence present on the chromosome. It turns out a lot of cancer-causing genes are present in ecDNAs. We’ve discovered that when a cancer-causing gene jumps off of the chromosome, it can make four times more message than the same sequence originally on the chromosome, so it gives the cancer cell a huge advantage. This is a problem that affects half of human cancer types.

*JCI*: Describe a recent or future project that you’re excited about.

Chang: We’re really interested in a class of RNAs that has a different kind of shape. When you think about RNAs, maybe think about little squiggles, like a line; that’s how they’re drawn in textbooks. There’s a new class of RNAs where the two ends are joined together. The RNA is a circle, and that’s called circular RNA. People joke that, in my lab we’re all going around in circles because the ecDNA is a circle; circular RNA is a circle. The circular RNAs were initially put in the noncoding RNA category, which is part of the reason why we were studying these. But we learned something interesting. It turns out that the way the body gets rid of the RNAs in cells and in bodies is to chew them up from the ends by enzymes. Circular RNA has no ends, so it lasts for weeks in the body rather than just a few hours. There are all sorts of potentially wonderful biotechnology applications from this new class of RNAs. For example, we can engineer a circular RNA and have it make a protein of interest, like a therapeutic protein. If you inject that once, the circular RNA will provide continuous protein production for a week or more. It is a potentially useful application. Another application involves using circular RNA to make a vaccine. It provides a very strong T cell response that could be used to fight cancer. Both of those are interesting directions that we’re following.

*JCI*: Tell us a little bit about the kind of mentorship you received and how it has influenced the way you mentor the next generation of scientists.

Chang: I was fortunate to have really thoughtful and supportive mentorship in my own training that fostered a lot of independence and support when needed. I try to foster that same kind of environment in my own group. One example is during our weekly lab meetings everybody presents weekly so that everybody knows what’s going on. And everyone gives feedback to other lab members’ projects. I want people to think beyond what they’re doing with their own hands and their own immediate project, but also to think about other kinds of science that’s going on. I think that’s going help them out a lot when they have their own groups because then they have much more experience. Once they have seen multiple projects progress step by step, not just the final results in the publication, they will understand all the pain points that somebody has to go through.

*JCI*: What does winning the Korsmeyer Award mean to you?

Chang: I’m really grateful for this recognition, especially for recognition of the mentorship. I’ve been fortunate to have received fantastic mentorship and I try to foster that and pass it onward. Probably the most important and lasting legacy for my career will be my trainees. Many of them are doing well and spawning off exciting science and mentoring their own students. So, I really appreciate the Korsmeyer Award for recognizing the twin aspects of a scientific career: discovery and mentorship. Of course, I’m a proud member of the ASCI and it’s especially meaningful to be recognized by my peers. I’m delighted by this honor.

## Figures and Tables

**Figure 1:**
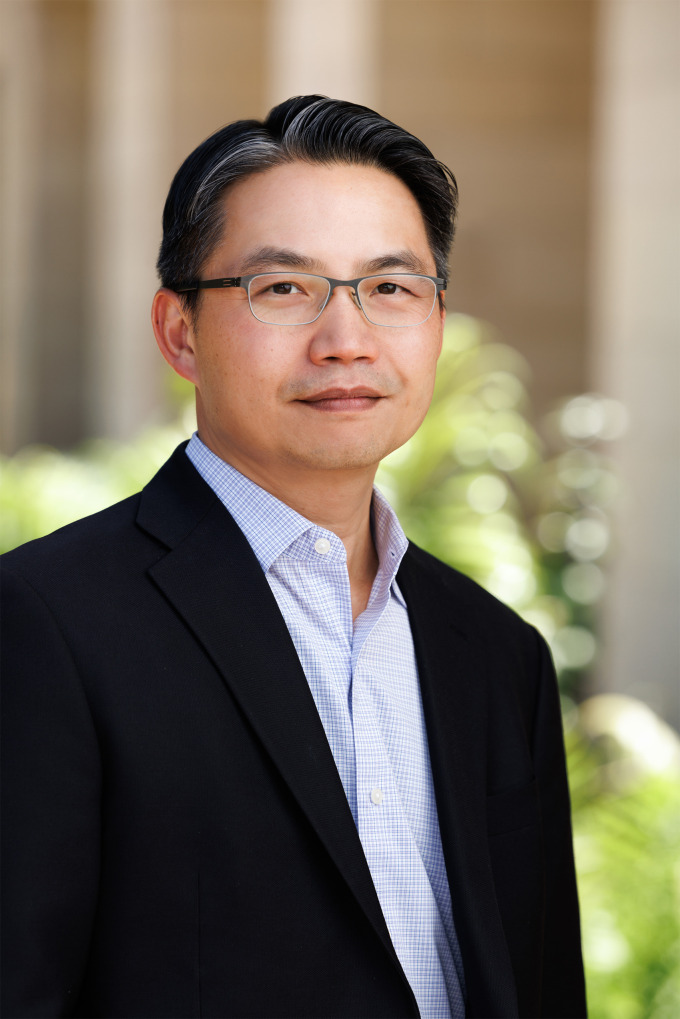
Howard Chang is the recipient 2024 ASCI/Stanley J. Korsmeyer Award.

## Version 1 04/01/2024

Electronic publication
